# Estimation of physiological genomic estimated breeding values (PGEBV) combining full hyperspectral and marker data across environments for grain yield under combined heat and drought stress in tropical maize (*Zea mays* L.)

**DOI:** 10.1371/journal.pone.0212200

**Published:** 2019-03-20

**Authors:** Samuel Trachsel, Thanda Dhliwayo, Lorena Gonzalez Perez, Jose Alberto Mendoza Lugo, Mathias Trachsel

**Affiliations:** 1 International Maize and Wheat Improvement Center (CIMMYT), Global Maize Program, Texcoco, Edo de Mex, Mexico; 2 International Maize and Wheat Improvement Center (CIMMYT), Sustainable Intensification Program, Ciudad Obregon, Sonora, Mexico; 3 University of Wisconsin, Department of Geography, Madison, Madison, WI, United States of America; International Rice Research Institute, PHILIPPINES

## Abstract

High throughput phenotyping technologies are lagging behind modern marker technology impairing the use of secondary traits to increase genetic gains in plant breeding. We aimed to assess whether the combined use of hyperspectral data with modern marker technology could be used to improve across location pre-harvest yield predictions using different statistical models. A maize bi-parental doubled haploid (DH) population derived from F1, which consisted of 97 lines was evaluated in testcross combination under heat stress as well as combined heat and drought stress during the 2014 and 2016 summer season in Ciudad Obregon, Sonora, Mexico (27°20” N, 109°54” W, 38 m asl). Full hyperspectral data, indicative of crop physiological processes at the canopy level, was repeatedly measured throughout the grain filling period and related to grain yield. Partial least squares regression (PLSR), random forest (RF), ridge regression (RR) and Bayesian ridge regression (BayesB) were used to assess prediction accuracies on grain yield within (two-fold cross-validation) and across environments (leave-one-environment-out-cross-validation) using molecular markers (M), hyperspectral data (H) and the combination of both (HM). Highest prediction accuracy for grain yield averaged across within and across location predictions (rGP) were obtained for BayesB followed by RR, RF and PLSR. The combined use of hyperspectral and molecular marker data as input factor on average had higher predictions for grain yield than hyperspectral data or molecular marker data alone. The highest prediction accuracy for grain yield across environments was measured for BayesB when molecular marker data and hyperspectral data were used as input factors, while the highest within environment prediction was obtained when BayesB was used in combination with hyperspectral data. It is discussed how the combined use of hyperspectral data with molecular marker technology could be used to introduce physiological genomic estimated breeding values (PGEBV) as a pre-harvest decision support tool to select genetically superior lines.

## Introduction

To meet the future demand of food, feed, fiber, and fuel, crop production must double by 2050 [1]. Crop yields are limited inherently by biotic and abiotic stresses, whereas plant researchers try to protect yield from plant stress losses by incorporating alleles that confer resistance to diseases and improving resistance to abiotic stresses resulting from changes in climate [2].

It was shown [1, 3] that each accumulated degree-day above 30°C reduced harvestable grain yield by 1% under optimal rain-fed conditions. It is therefore crucial to develop germplasm able to cope with anticipated climate change scenarios to provide sufficient food in the future and to increase genetic gain towards that goal [1]. Modern breeding tools and methods in genomics and genetics (e.g. next generation sequencing) have tremendously helped to reduce experiment cost and make the genomic technologies available for routine use in most corporate crop improvement programs. Modern plant breeding tools such as marker-assisted selection (MAS) and genomic selection (GS) have been shown to improve genetic efficiency for selection of both qualitative and quantitative traits as compared to phenotypic selection alone [4, 5, 6]. By simultaneously estimating all marker effects as done with GS, variation can be captured that may otherwise not be detectable using traditional statistical approaches [7]. With GS, a training set that has been phenotyped and genotyped, should be used to calibrate a prediction model, which is then used to predict the genomic estimated breeding values (GEBV) of a ‘test set’ of genotyped selection candidates [8]. To successfully make use of GS, it is critical that the training populations be phenotyped with high accuracy to establish reliable marker phenotype relationships in order to predict non-tested genotypes. Unfortunately, current phenotyping technologies are still lagging behind and limiting the use of modern marker technology.

In addition to grain yield secondary traits could be used to increase selection intensity. Selection on secondary traits is beneficial when the secondary trait is highly heritable, highly genetically correlated with the target trait, also if this secondary trait is cheaper or easier to measure than the target trait [9]. Utility of secondary traits is typically environment dependent [10], which makes indirect selection challenging. Multivariate models overcome this problem because genetic covariances among traits are estimated using a model training set that is representative of the selection candidates and evaluated in the target environment(s). Multivariate models including secondary traits have been shown to increase prediction accuracy and reduce bias as compared to univariate models, when secondary traits are measured in both the model training and testing population [11,12]. Variation in foliar reflectance at different wavelengths in the spectrum is specific to variation in different, chemical and structural components of leaves (e.g. chlorophyll, anthocyanins and water content [13,14]). Therefore, analysis of foliar reflectance spectra has the potential to rapidly assess multiple physiological and biochemical traits from a single measurement [15]. Many of the physiological and agronomic traits of a crop that influence grain yield also lead to differences in the reflectance of electromagnetic radiation at different wavelengths (e.g. chlorophyll content, leaf greenness, canopy water mass content [16, 17]). The evolution of remote sensing over the past two decades (1998–2018) has allowed for the quantification of differences in leaf area (measured as NDVI) or leaf chlorophyll content (measured as GRE) among genotypes and different agronomic management (e.g. irrigation or nitrogen fertilization treatments) using spectral data, providing insights on different aspects of crop physiology. Remote sensing (Aircraft, UAV or Satellite based systems) has been used in plot management, while blimps [18], aircraft [19, 20] or UAV based multispectral and hyperspectral cameras have been used to measure multiple crop indices at the plot level in plant breeding (e.g. CWMI [17], NDVI [17, 18, 19, 20]; canopy temperature [18, 19]). Basic agronomic traits such as stand counts and lodging are routinely measured in breeding/testing programs using small UAVs provided by multiple commercial providers (e.g. Delair, Labege, France, http://delair.aero, last visited January 2019; Precisionhawk, Raleigh, NC, USA, http://www.precisionhawk.com; last visited January 2019). A promising technology to facilitate data-driven breeding by capturing relevant (physiological) crop information are UAV mounted hyperspectral cameras. Full spectral information acquired with hyperspectral cameras used in combination with Bayesian mathematics outperformed currently used composite indices for grain yield prediction under abiotic stress [17]. It was furthermore shown [17], that the closer the measurement was to harvest the higher the prediction accuracy was for grain yield, reaching a maximum (~0.5) when data from five measurements taken after flowering were combined. In computational biology, the analysis of data sets containing tens of thousands of features (“large p”), but only a few hundred samples (“small n”), is nowadays routine, and several regression and machine learning approaches such as partial least squares (PLSR), random forest (RF), ridge-regression (RR) and Bayesian ridge regression (BayesB) are popular choices in recent literature.

Partial least square regression is one of the least restrictive extensions of the multiple linear regression model allowing it to be used in situations where the use of traditional multivariate methods is severely limited, such as when there are fewer observations than predictor variables. Random Forest is a highly data adaptive supervised classification algorithm, that is able to account for multicollinearity and interactions among features making random forest appealing for high-dimensional (genomic) data analysis [21]. However, random forest(s) tend to overfit models, they are computationally intensive, and are difficult to interpret since one can neither see nor understand the relationship between the response and independent variables. Ridge regression regularizes coefficients allowing the use of complex models while avoiding over-fitting. It accounts for multicollinearity among predictor variables by adding a degree of bias to regression variables. It is one of the most popular algorithm used for genomic selection in plant breeding literature [20, 22, 23]. Like ridge regression, Bayesian regression techniques use regularization parameters in the estimation procedure. In contrast to ridge regression, the regularization parameter is not set in a hard sense but adapted to the structure of parameters and genotypic values. These priors induce a type of shrinkage of estimates that is conditional on the effect size of a marker/input parameter [22]. Unlike ridge regression, Bayesian methods can set markers with little/no effects to zero. Depending on marker type used and architecture of the trait evaluated BayesB is expected to perform similarly to ridge regression [23, 24]. However, it is typically more computationally intensive than ridge regression.

Depending on individual breeding programs, intrinsic cut off dates throughout the growing season (or off season) may include the selection of parents for new population starts, prioritization of families to be sent for doubled-haploid induction or lines to be used for hybrid make up without the availability of complete information on lines involved. Accurate pre-harvest yield estimates would therefore be useful for breeders for various purposes when making critical decisions when only limited (yield) data is available. In the present study it is hypothesized that combining high density marker information with reflectance data using machine learning algorithms would further improve prediction accuracies and GEBVs of grain yield estimates pre-harvest across environments.

The main objectives of this study were to assess i) whether pre-harvest predictions of grain yield could be improved across environments when molecular markers are combined with hyperspectral data and ii) to identify the most suitable statistical method to maximize prediction accuracy.

## Materials and methods

All trials of this study were carried out in agreement with landowners (CIMMYT) owning the land used for these trials. Crop management (agronomy) and phenotyping did not have any adverse effects on the natural environment. Crop management treatment (well-watered vs drought stressed) and phenotyping did not have any adverse effect on land outside the trial area.

### Germplasm

A maize bi-parental DH population, consisting of 97 F1 derived lines, was evaluated under heat stress as well as combined heat and drought stress. The maize DH population used, was developed from the F1 of the cross between two yellow lines: CML451 and DTPYC9F46. The parental inbred line DTPYC9F46 was specifically selected for tolerance to drought [25] and has been used as source germplasm for drought and heat tolerance. Whereas the other parental line CML451 was an elite inbred line selected for yield potential and disease tolerance. Doubled haploid lines were further crossed to CL02450 to form testcross hybrids for evaluation in this study.

### Trial management

Trials were carried out under heat (maximum day temperatures > 35°C around flowering and during grain filling) and combined heat and drought stress at CIMMYT’s experiment station in Ciudad Obregon, Sonora, Mexico (27°20” N, 109°54” W, 38 m asl), during the summer season in 2014 and 2016. Trials were planted on June 20 in 2014 and May 31 in 2016 and harvested October 7 2014 and September 29 2016, respectively. The experiments were planted in single row plots 4.5 m long at a population density of 6.9 plants m^-2^, with 80 cm between rows. Trials were laid out in an α-lattice incomplete block design replicated twice. All trials received two fertilizations: 100 kg ha^-1^ of (NH_4_)H_2_PO_4_ and 500 kg ha^-1^ (NH_4_)_2_SO_4_ at sowing and 250 kg ha^-1^ of (NH_4_)_2_SO_4_ at V5 [25]. The treatment combining heat and drought stress was fully irrigated up to ~750 GDD after planting (12–15 d before anticipated flowering). Thereafter, irrigation was reduced to 50% of relative potential evapotranspiration. Irrigation was applied twice weekly using drip irrigation at a rate of 5 mm h^-1^ for 6 to 14 h depending on potential evapotranspiration. Irrigations were corrected when water was more readily available from rainfall. Weeds, insects, and diseases were controlled as needed.

### Acquisition and processing of hyperspectral images

Image data were collected using a hyperspectral camera (VNIR Headwall Photonics Micro-Hyperspec ARS3, Headwall Photonics) mounted on a single-engine aircraft Piper PA-16 Clipper. Flights started 55 d after sowing (when most plots had 50% of plants flowering; R1 stage [26]) and were repeated at 62, 69, 75, and 83 d after sowing (hereinafter labeled as F1, F2, …, and F5, respectively). To achieve a resolution of 30 cm pixel^-1^, flights were performed at an altitude of 300 m and a ground speed of ~34 m s^-1^. The hyperspectral camera had a radiometric resolution of 10 bits. It acquired images from 392 to 850 nm, subdivided into 62 evenly spaced bands at a spectral resolution of 1.9 nm, covering the visible spectrum and part of the near infrared (NIR) spectrum. A filter was applied to the images to exclude pixels corresponding to a mixture of crop and soil, and to calibrate reflectance intensity. The atmospheric correction was performed with the SMARTS simulation model developed by the National Renewable Energy Laboratory of the USDOE [27]. This was done using aerosol optical depth measured at 550 nm with a Micro-Tops II sun photometer (Solar LIGHT Company). The hyperspectral camera was radiometrically calibrated with a uniform light source system (integrating sphere, CSTM-USS-2000C Uniform Source System, LabSphere) at four different levels of illumination and six different integration times. Plot images were trimmed by excluding borders of two to three pixels per plot. The plot coordinates were defined based on a grid of polygons representing the trial plots. This grid was adjusted on the map based on the actual location of certain plots in the field, measured with a Trimble R4 GPS receiver. Each of the 62 reflectance bands was measured using a mean value obtained from the central plot pixels.

In addition to spectral data, plant height, anthesis, silking and grain yield were measured. During flowering, the number of days from the planting date by which 50% of plants within a plot were shedding pollen and growing silks were recorded as anthesis (AD) and silking (SD) dates, respectively. The anthesis silking interval (ASI) was calculated as the difference between silking and anthesis. Plant height was measured two weeks before harvest as the distance from ground level to the flag leaf. Plants were hand harvested when all plots had <15% grain moisture. Ears harvested from each plot were shelled, weighed, and subsampled for measuring grain moisture. The trait analyzed in this study was grain yield adjusted to 12.5% moisture and converted to metric tons per hectare.

### Linkage map

Genomic DNA was isolated from young leave tissue using a CTAB procedure (CIMMYT Applied Molecular Genetics Laboratory 2003). DNA of all the samples was sent to Cornell University Biotechnology Resource Center (Ithaca, NY, USA). Genomic DNA from each sample were digested with *ApeKI* enzyme (New England Bio-labs, Ipswich, MA), constructed 96-plex GBS libraries and sequenced by Illumina HiSeq2000 (Illumina Inc., San Diego, CA, USA). TASSEL GBS Pipeline was used for high-quality single nucleotide polymorphisms (SNPs) calling. GBS 2.7 TOPM (tags on physical map) file was downloaded from Panzea (www.panzea.org), and it was used to anchor reads to the reference genome Maize B73 RefGen_v2 [28]. Un-imputed GBS dataset were used for further analyses in the bi-parental populations.

A bin map was constructed using high quality un-imputed SNPs with customized R scripts [28]. In order to reduce genotyping error and eliminate the low quality SNPs from the bin map, the following steps were performed: (1) un-imputed SNP datasets were filtered with the parameters of minor allele frequency greater than 0.05 and missing rate less than 20%; (2) DH lines with heterozygosity rate greater than 5% and/or missing rate greater than 20% were eliminated from the further analysis; (3) unlinked SNPs were removed from further analysis, where the window size was 8, similarity rates of all the SNPs within each window were calculated to remove the unlinked SNPs, threshold of similarity rate was 95%; (4) the consecutive SNPs with high similarity rate, i.e., 95%, were merged into one bin; and (5) bins were treated as genetic markers to construct a genetic map. 27818 SNPs were clustered into 494 bins and the genetic map length was 1150.16 cM. Genetic map for each population was built with software QTL IciMapping Version 4.0 (www.isbreeding.net) as described earlier [29].

### Phenotypic data analysis

Phenotypic data were analyzed using the following linear mixed model [30]:

$$Y_{mhlk}=u+a_{h}+E_{ml}+a_{h}E_{ml}+r(E_{ml})+r(E_{ml})\delta_{k}+\varepsilon_{mhlk}$$ (1)

where Y_*mhlk*_ is the trait value of the h^th^ genotype (h = 97) for the 1^th^ environment (1 = 4), defined as treatment-by-year combination and the m^th^ replication (m = 2); u: the overall mean, a_*h*_: the main effect of the genotype, E_*ml*_: the effect of the environment, a_*h*_E_*ml*_: the genotype-by-environment interaction, r(E_*ml*_): the replication within environment effect; r(E_*ml*_)δ_*k*_: the effect of blocks within replicates within environments and ε_*mhlk*_: the error term. All factors were set as random factors for the estimation of variance components, while the factor genotype was set as fixed effect to estimate best linear unbiased estimators (BLUEs) within each environment.

Best linear unbiased estimators (BLUEs) within environment and broad-sense heritability were calculated using META-R Version 5.0 [30]. The repeatability (h^2^) was estimated with a method described previously [31]. Variance components were estimated by restricted maximum likelihood (REML) and repeatability as the relationship between genetic and phenotypic variance, per the formula:

$$h^{2}=\frac{{\sigma_{G}}^{2}}{{\sigma_{G}}^{2}+(\frac{{\sigma_{GxE}}^{2}}{l})+\frac{e}{r*l})}$$ (2)

where *σ_G_*^2^ is the genotypic variance, *σ_GxE_*^2^ the genotype-by-environment interaction variance, l the number of environments and r the number. An environment was defined as unique season-by-irrigation treatment combination.

#### Inclusion of markers and hyperspectral values into the statistical model

Input parameters used were molecular markers, (hyperspectral) reflectance data from 62 bandwidths measured at five points in time after flowering and the combination of both marker and (hyperspectral) reflectance data. All models were fit in a two-step process: first calculating BLUEs for each genotype and trait based on measured phenotypic data (as described above), and second fitting the prediction model with the calculated BLUEs for individual bandwidths and/or genomic markers as input variables and grain yield as target variable.

Different univariate models were used to predict grain yield. The models included the BLUE for grain yield (GY) as target variable, the overall mean, effects for molecular markers and/or individual bandwidths measured with the hyperspectral camera as input variables used as random matrix and the random error term e. The matrix used, contained marker information only (marker model), BLUEs for unique genotype-by-bandwidths-by-time point combinations measured with the hyperspectral camera (Hyperspectral model only) or the combination of both the marker and the hyperspectral information (H+M model).

#### Within and across environment predictions

In order to predict germplasm within environment datasets were equally and randomly split in a training and a test set. The training set was used to parametrize the statistical model, while the test set was predicted. Regressions coefficients were estimated using either partial least square regression (PLSR), random forest (RF), ridge regression (RR) or BayesB. A Bayesian shrinkage-variable selection procedure using a prior with a point of mass at zero and a t-slab was used for BayesB. Due to differential computation time/requirement among statistical procedures 2-fold cross validation was performed with a different number of replications: 1000 times for PLSR, 2000 times for RF, 3000 times for RR and 1000 times for BayesB.

For across environment predictions all entries within three environments were used to predict entries in the fourth environment using a leave-one-environment-out cross-validation. The standard errors of correlation estimates were estimated using a bootstrap procedure with 10,000 replicates. All within and across environment prediction for all leave-one-out-combinations were used to get an average for the reported prediction accuracy. For cross-validation schemes, the Pearson correlation between the predicted values of the model and the observed BLUE value for GY were used as a measure of prediction accuracy (r_GP_).

#### Estimation methods and Software

All analyses were performed using R software (R version 3.4.4; [32]). Partial least square regression, random forest, ridge regression and Bayesian ridge regression were implemented using the pls [33], random forest [34], rrBLUP [35] and BGLR [36] packages, respectively. The average value of the Pearson correlation coefficients [32] between the phenotype and the predicted values was defined as prediction accuracy (r_GP_). It was calculated using the cor.test function in R [32].

## Results

### Genetic map

The initial un-imputed GBS data included 955690 SNPs for all the DH lines; 955120 of them were evenly distributed on chromosomes 1 to 10, and the number of SNPs on each chromosome ranged from 148752 on chromosome 1 to 67126 on chromosome 10. After filtering with minor allele frequency (MAF) greater than 0.05 and missing rates less than 20%, the total number of SNPs decreased to 47203. After filtering, the missing rate decreased from 42.32% to 7.90% while the heterozygosity rate increased from 0.47% to 2.55%. After filtering, the average MAF was 0.42 and 79.74% of the SNPs concentrated to the MAF ranging from 0.40 to 0.50.

### Environmental variables

**Fig 1:**
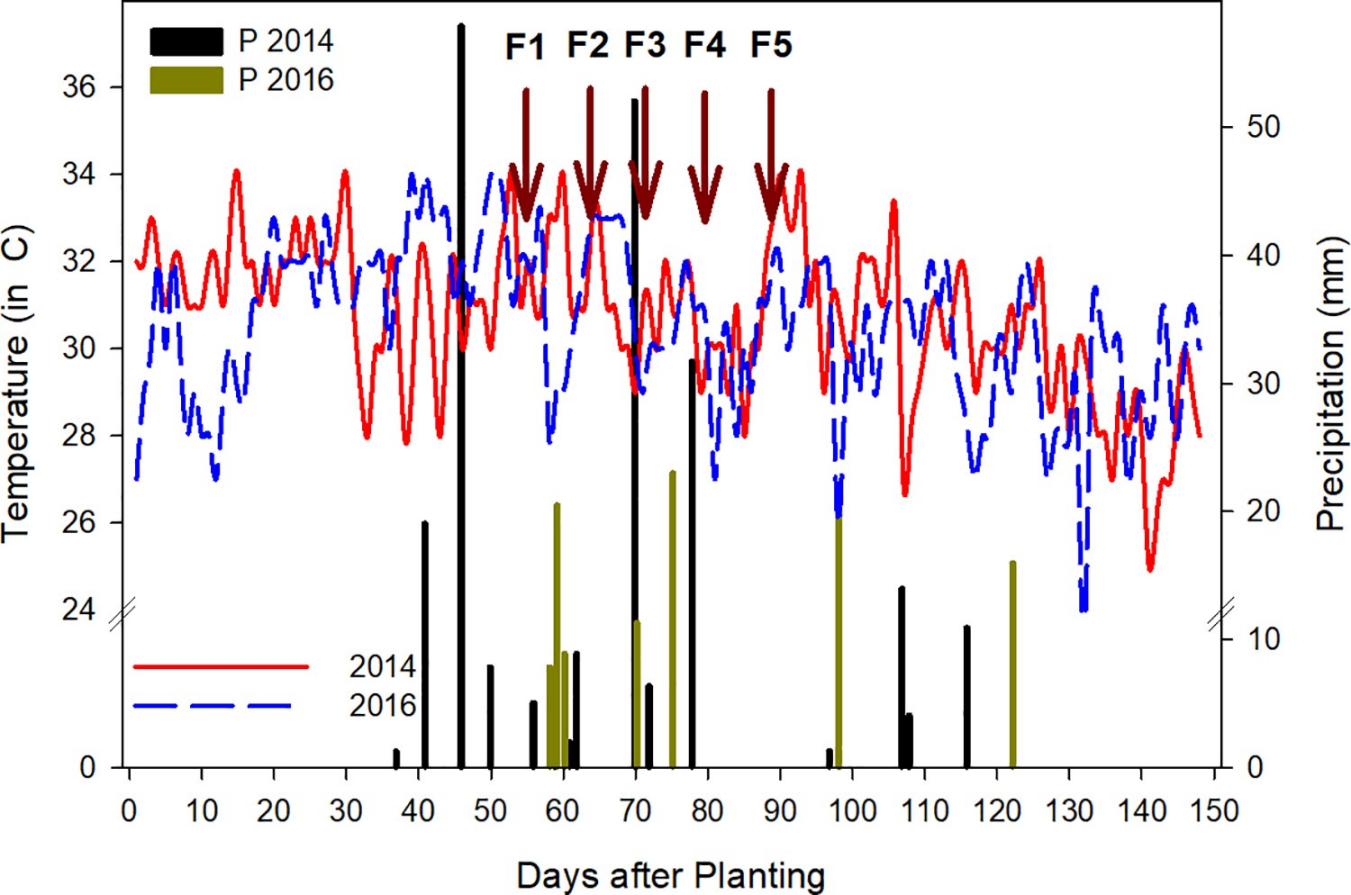
Average daily distribution of temperature and precipitation for trials carried out in the summer of 2014 (temperature: solid red line; precipitation: black bars) and 2016 (temperature: dashed blue line; precipitation: green bars) under well-watered and drought stressed conditions in Ciudad Obregon (Sonora, Mexico) relative to the planting date. Arrows indicate date of five flights starting at flowering (F1 to F5). Flights took place 55 (F1), 62 (F2), 69 (F3), 75 (F4) and 83 (F5) days after planting.

Temperatures were comparable across cropping season in both years reaching an average of 29.1°C in 2014 and 28.9°C in 2016 (Fig 1). Average temperatures during emergence/pre-flowering were 1.6°C higher in 2014 (32.0°C) relative to 2016 (30.4°C), while daily mean temperatures around flowering were similar in 2014 (31.4°C) and 2016 (31.5°C), respectively. Multiple strong rains around flowering in 2014 (80 mm relative to 28 mm in 2016) resulted in lower stress levels around flowering and slower dry down.

### Phenotyping of yield and agronomic traits

**Fig 2:**
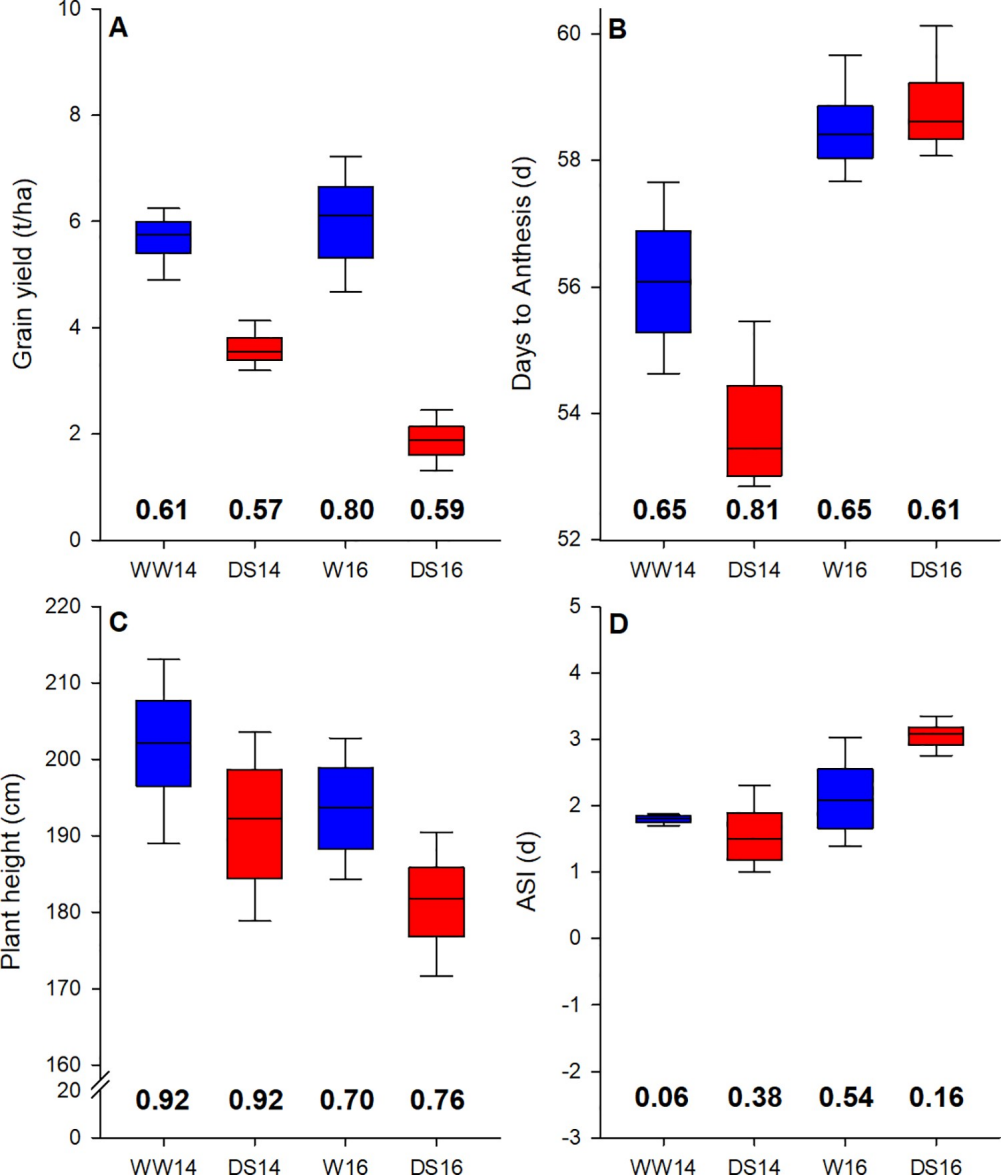
Distribution of trait values and repeatability for trials carried out under well-watered (blue boxplots) and drought stressed (red boxplots) conditions for trials carried out in 2014 and 2016. Phenotypic traits shown are grain yield (A), days to anthesis (B), plant height (C) and the anthesis silking interval (ASI; D).

The analysis of variance showed that genotype, environment, and genotype-by-environment interaction were highly significant (P<0.01) for grain yield, the anthesis silking interval, plant height, and AD. Most spectral bandwidths were equally affected by factors genotype, environment and the interaction between both (P< 0.05; Fig 2). Grain yield under well-watered conditions was comparable in 2014 (WW: 5.7 Mg ha^-1^; DS: 3.6 Mg ha^-1^) and 2016 (WW: 5.9 Mg ha^-1^; DS: 1.9 Mg ha^-1^). The fact that reductions under drought stress relative to the well-watered treatment were less accentuated in 2014 (-2.1 Mg ha^-1^ in 2014 vs -4 Mg ha^-1^ in 2016) can potentially be attributed to differences in precipitation in the 10 day (+-5 days) period bracketing flowering (80 mm in 2014 relative to 28 mm in 2016). This difference in precipitation might also explain the larger anthesis silking interval under drought stress in 2016 (3.1 d) relative to 2014 (1.6 d) indicative of greater stress levels around flowering in 2016. Higher temperatures pre-flowering could potentially explain why plants flowered earlier in 2014 (55 d) relative to 2016 (58 d). Trait repeatability ranged from 0.06 (anthesis silking interval in 14WW) to 0.92 (plant height in 14DS), indicative of high data quality for the trials.

### Hyperspectral data

**Fig 3:**
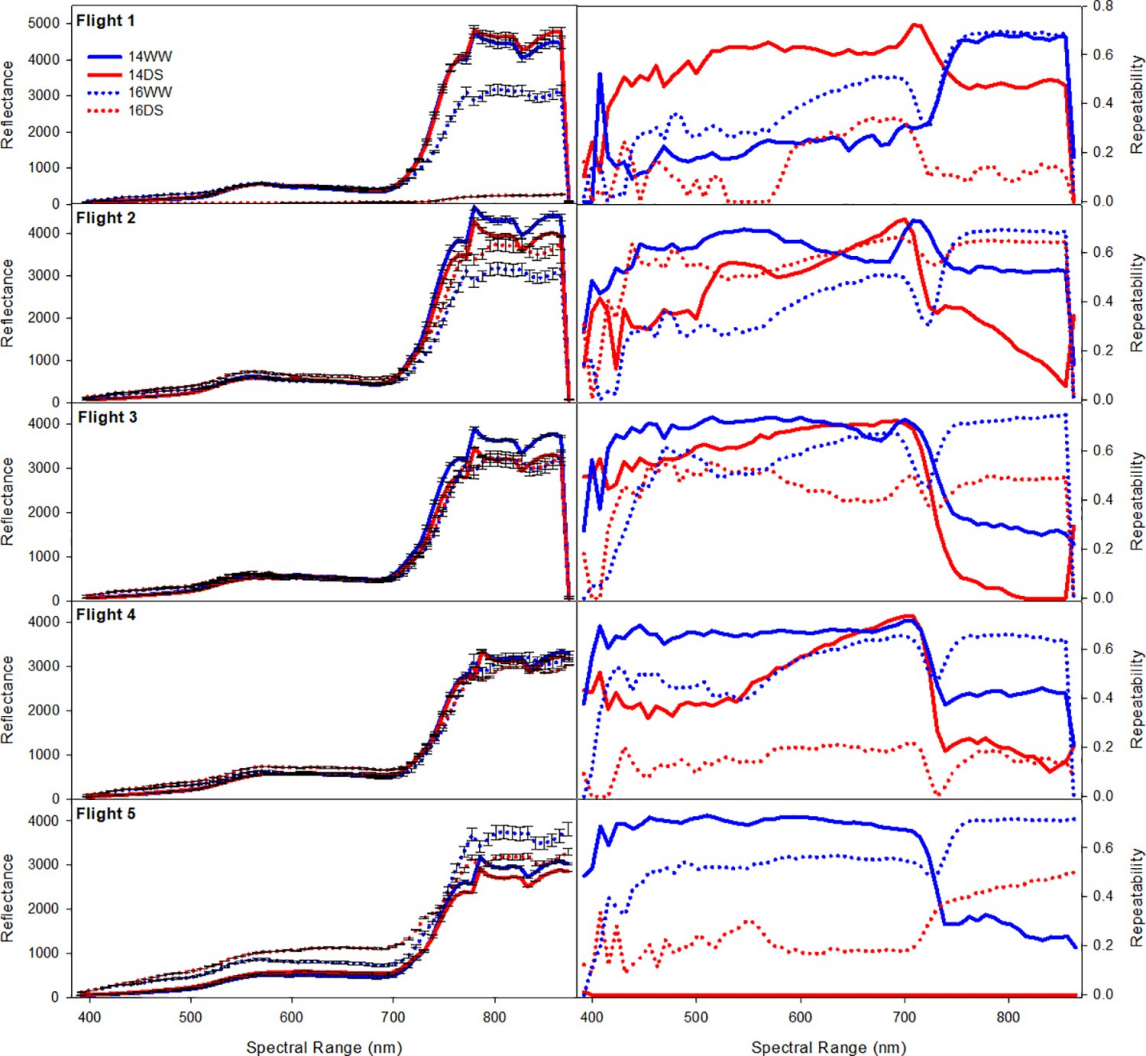
Reflectance (left column) and repeatability for individual bandwidths (right column) across the spectral range from 392 nm to 850 nm for individual flights in the well-watered (blue line) and drought stressed (red line) treatment in 2014 (solid line) and 2016 (dotted line).

Very little differentiation among genotypes or treatments was observed in the range of visible light between 400 and 700 nm. Above 700 nm (in the red and infrared range) clear differences among treatments and genotypes could be ascertained (Fig 3).

Repeatability for individual wavelengths measured with the hyperspectral camera varied across environments (14WW, 16WW, 14DS, 16DS) and spectral range (392–850 nm). Averaged across treatments repeatability was moderately higher for 2014 trials (h^2^ = 0.45) relative to trials carried out in 2016 (h^2^ = 0.41). Averaged across years the WW treatment (h^2^ = 0.52) had a higher repeatability relative to the drought stressed treatment (h^2^ = 0.34). Interestingly repeatability was higher in the bandwidths in the visual range in 2014 (h^2^ = 0.45) relative to 2016 (h^2^ = 0.36), while repeatability for bandwidths in the red/ infrared (IR) range was higher in 2016 (h^2^ = 0.48) relative to 2014 (h^2^ = 0.36) indicative of greater stress levels in 2016 which allowed for better differentiation among treatments and genotypes in the red/IR range (Fig 3).

### Prediction accuracy

**Fig 4:**
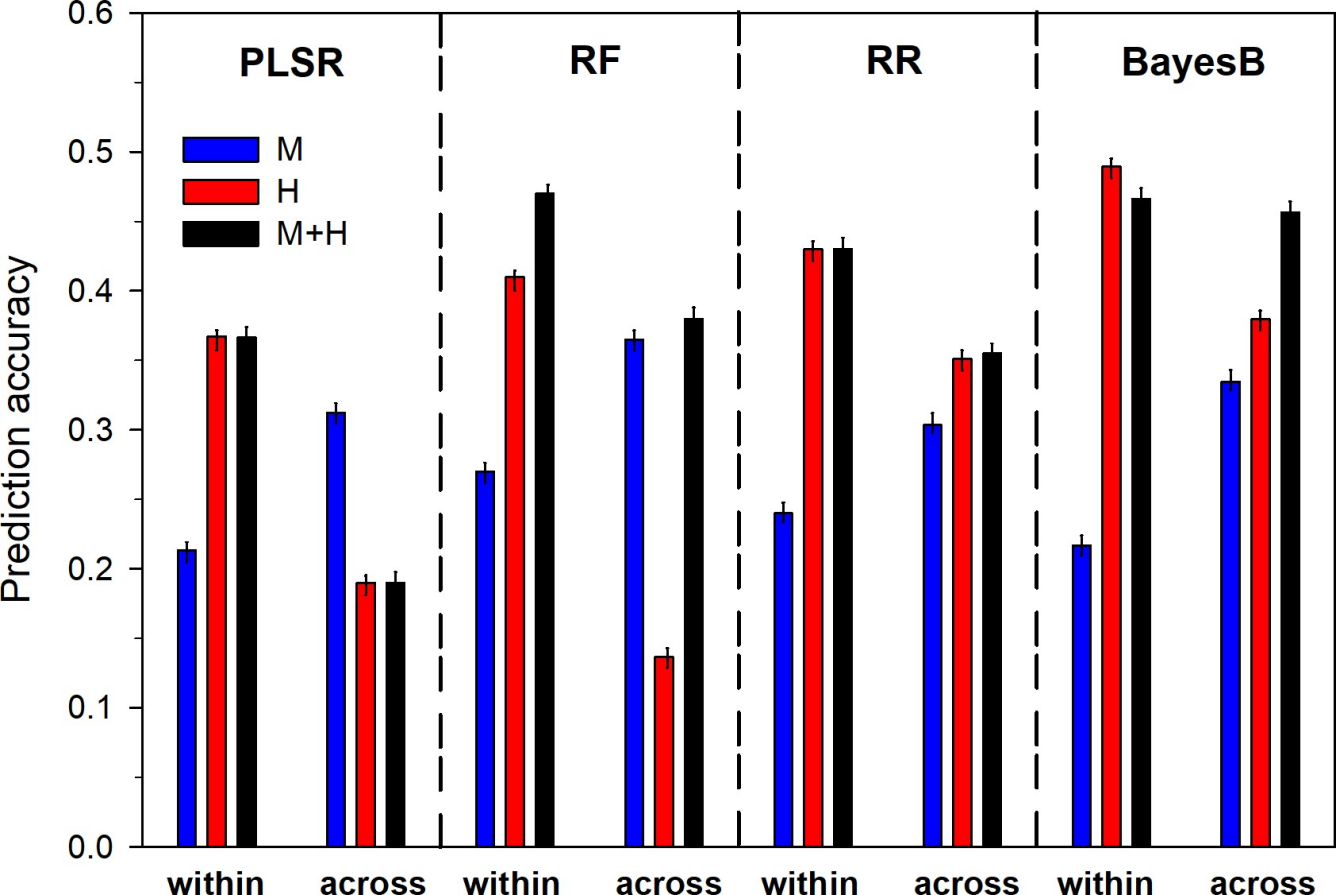
Prediction accuracy for within and across environment predictions using different statistical methods and input parameters. Statistical methods used were partial least square regression (PLSR), random forest (RF), ridge regression (RR) and Bayesian ridge regression (BayesB). Input factors used for model parametrization were molecular markers (M), hyperspectral reflectance data (H) and the combination of both (HM).

The current manuscript evaluated the effects of different statistical models (PLSR, RF, RR and BayesB) and input factors (molecular markers, hyperspectral data, combination of molecular markers and hyperspectral data) on prediction accuracies within and across environments for grain yield. Overall, r_GP_ ranged from 0.14 (across environments using random forest; Fig 4) to 0.49 (within environments using BayesB). The highest prediction accuracy across environments (r_GP_ = 0.47) was measured for BayesB when molecular marker data and hyperspectral data were used as input factor, while the highest within environment prediction (r_GP_ = 0.49) was obtained when BayesB was used in combination with hyperspectral data. In agreement with lower within environment variance, the r_GP_ was generally higher within (r_GP_ = 0.36) than across environments (r_GP_ = 0.31).

Among the statistical models used BayesB (r_GP_ = 0.39), averaged across within and across location predictions, had highest r_GP_ followed by ridge regression (r_GP_ = 0.35), random forest (r_GP_ = 0.34) and partial least square regression (r_GP_ = 0.27), confirming the utility of general parameter shrinkage (as used in ridge regression) or Gaussian parameter shrinkage (as used in BayesB) for model calibration and prediction. Using the combination of hyperspectral and marker data (r_GP_ = 0.39) as input factor on average yielded better predictions than using marker data (r_GP_ = 0.28) or hyperspectral data (r_GP_ = 0.34) only. Depending on input factors and model combinations deviations from this general pattern were observed.

For within location predictions, combining marker and hyperspectral data did not yield higher r_GP_ when using partial least square regression (r_GP_ = 0.37) and ridge regression (r_GP_ = 0.43) and even resulted in lower prediction accuracy relative to using hyperspectral data only with BayesB (H: r_GP_ = 0.49 vs HM: r_GP_ = 0.47), It did neither add any benefit for across environment predictions (H: r_GP_ = 0.35, HM: r_GP_ = 0.35) when ridge regression was used. Low r_GP_ across environments using random forest for hyperspectral data or the combination of markers and hyperspectral data is related to low r_GP_ for random forest when predicting the 2014 environments.

## Discussion

High quality trials with reasonable heat and drought stress across years and irrigations treatments as indicated by yield reductions relative to non-stressed trials, average daily temperatures above 32º C and high data repeatability, were established. Reductions in grain yield in response to drought stress, were in the range of what is typically measured in such trials [19, 37]. Unexpected rains (80 mm in 2014 relative to 28 mm in 2016) around flowering resulted in lower stress levels in 2014 drought trials and a significant genotype-by-environment interaction.

Wavelengths of 400 nm to 700 nm allowed little genotypic differentiation among treatments and years as reported previously for similar studies [20, 38]. Wavelengths in the red/IR range clearly differentiated among genotypes and induced environmental conditions re-emphasizing the importance of this range for stress detection as suggested previously [38, 39]. In addition to data (partially) presented earlier [17], molecular marker data and an additional season of data was added to the analysis in this study. Since it was shown [17] that prediction accuracy for grain yield was highest when data from multiple timepoints was used, the current study does not focus on data from individual measurements for grain yield predictions. The combined use of marker and hyperspectral information (r_GP_ = 0.39 averaged for across and within environments) predicted better than markers (r_GP_ = 0.28) or hyperspectral (r_GP_ = 0.34) data alone for within and across environments averaged across statistical methods. The difference was most accentuated for ridge regression (M: r_GP_ = 0.3 vs HM: r_GP_ = 0.35) and BayesB (M: r_GP_ = 0.33 vs HM: r_GP_ = 0.46). Both ridge regression and BayesB allow to regularize (estimate) coefficients for robust (complex) models. While ridge regression sets the regularization parameter in a hard sense, BayesB adapts the regularization parameter to the data at hand using a priori assumptions conditional on the marker-by-phenotype relationship resulting in a shrinkage of estimates [23]. Adaptive to data, while not overfitting makes them superior to random forest and partial least square regression. As a result of their robustness and versatility they were successfully used for predictions using genomic [6] or spectral information (BayesB[18]) or the combination of both [40, 41, 42] in the past. Simulation studies [43] furthermore confirmed that BayesB outperformed ridge regression if there are only few QTL, as was indeed the case here (unpublished data) as expected for a highly quantitative trait like grain yield under abiotic stress.

Prediction accuracy (r_GP_) for the combination of marker and hyperspectral data as well as for hyperspectral data alone dropped when using partial least squares and for hyperspectral data when using random forest for across environment predictions. The 2014 environments predicted poorly (r_GP_: -0.1 to 0.1,) because of the observed genotype-by-environment interaction, the intrinsic characteristic of overfitting of random forest models and to some extent for partial least square regression as observed previously [33, 44]. Although irrigation treatments applied (well-watered or drought stressed) were similar across years, resulting environmental conditions were completely different, i.e. none of the environments included for model training resembled the environmental conditions encountered in 2014 under combined heat and drought stress. Potentially overfitted random forest or partial least square models did therefore not have any factual foundation to predict these completely different data, as observed in earlier studies [33, 34, 43, 45]. At the same time using markers or markers in combination with hyperspectral data allowed random forest to establish sufficient “genetic common ground” across environments to be able to provide decent prediction accuracy (M: r_GP_ = 0.36; HM: r_GP_ = 0.38). Evaluation of additional populations of different structure will be needed to determine how the small number of lines and the genetic structure of the evaluated double haploid population affected prediction accuracy.

Genomic estimated breeding values typically include information of multiple environments (years, locations, treatments) and multiple generations of trait values for specific genotypes. Hyperspectral information presented here could be used to improve the quality and accuracy of GEBVs by adding an additional layer of information on crop physiology.

While subjective information approximating physiological process have been used in the past (e.g. visual senescence scores [10]) the use of hyperspectral cameras in combination with an UAV would tremendously increase the throughput and increase objectivity of measurement. Wavelengths measured with a hyperspectral camera provide information on different physiological and biochemical processes [46] such as canopy water content, photosynthetic activity, leaf greenness, soil cover, leaf area index, canopy architecture and general plant status [20, 39]. Wavelengths measured with the hyperspectral camera therefore provide direct information on the biochemical and physiological status with direct effects on harvestable grain yield. Combining marker information with spectral data should therefore give a more accurate physiological GEBV (PGEBV) containing additional information determining yield formation before harvest.

Full genotypic information on germplasm in a breeding program is often only available for line and hybrid advancements after harvest. However, breeders need to make well founded (data based) decisions for various purposes throughout the year when only limited data is available. Depending on individual breeding programs, intrinsic cut off dates throughout the growing season (or off season) may include the selection of parents for new population starts, prioritization of families to be sent for doubled-haploid induction or lines to be used for hybrid make up without the availability of complete information on lines involved. Upon validation of this concept combining hyperspectral information with molecular information in a broader set of germplasm it will facilitate the breeding workflow as a pre-harvest decision support tool to select genetically superior lines.
